# Efficient Video Panoramic Image Stitching Based on an Improved Selection of Harris Corners and a Multiple-Constraint Corner Matching

**DOI:** 10.1371/journal.pone.0081182

**Published:** 2013-12-04

**Authors:** Minchen Zhu, Weizhi Wang, Binghan Liu, Jingshan Huang

**Affiliations:** 1 College of Mathematics and Computer Science, Fuzhou University, Fuzhou, Fujian, China; 2 College of Civil Engineering, Fuzhou University, Fuzhou, Fujian, China; 3 School of Computing, University of South Alabama, Mobile, Alabama, United States of America; University of Adelaide, Australia

## Abstract

Video panoramic image stitching is extremely time-consuming among other challenges. We present a new algorithm: (i) Improved, self-adaptive selection of Harris corners. The successful stitching relies heavily on the accuracy of corner selection. We fragment each image into numerous regions and select corners within each region according to the normalized variance of region grayscales. Such a selection is self-adaptive and guarantees that corners are distributed proportional to region texture information. The possible clustering of corners is also avoided. (ii) Multiple-constraint corner matching. The traditional Random Sample Consensus (RANSAC) algorithm is inefficient, especially when handling a large number of images with similar features. We filter out many inappropriate corners according to their position information, and then generate candidate matching pairs based on grayscales of adjacent regions around corners. Finally we apply multiple constraints on every two pairs to remove incorrectly matched pairs. By a significantly reduced number of iterations needed in RANSAC, the stitching can be performed in a much more efficient manner. Experiments demonstrate that (i) our corner matching is four times faster than normalized cross-correlation function (NCC) rough match in RANSAC and (ii) generated panoramas feature a smooth transition in overlapping image areas and satisfy real-time human visual requirements.

## Introduction

Video panoramic image stitching is based on the similarity of overlapping regions between adjacent images. State-of-the-art image registration (a.k.a. image alignment) algorithms can be classified into three different categories: intensity-based (for example, [1], [2]), frequency-domain-based (for example, [3]), and feature-based (for example, [4]–[11]). Intensity-based algorithms usually involve a large amount of computation and do not handle well the image alignment after rotation and scaling. Algorithms based on the frequency domain are in general faster and more appropriate for small translation, rotation, and scaling, but their performance is degraded when dealing with smaller overlapping regions. Therefore, more research has been focused on feature-based algorithms, which make use of a small number of invariant points, lines, or edges to align images. The computational complexity is reduced due to less information needing to be processed. In addition, they are robust to changes in image intensity. However, two issues have been identified for many feature-based algorithms. (i) Alignment outcomes are vulnerable to numerous factors (e.g., noise, information distribution pattern in images, and so forth) and result in a relatively low accuracy. In the area of video panorama stitching, more often than not, the overlapping between adjacent images is relatively small. Therefore, the successful stitching relies heavily on the accuracy and robustness of selected features. (ii) Many existing algorithms make use of an exhaustive search based on template matching. The computation, although already decreased to some extent, is still intensive, which does not meet the real-time requirement usually found in video panorama stitching.

Based on the above analysis on existing methodologies, we herein present a new algorithm. Our algorithm is classified as a feature-based algorithm and is motivated by the need to stitch adjacent images in both cases of (i) when there exist small overlapping areas and (ii) when their difference in translation, rotation, and scaling is small. Our algorithm has two preferable features, i.e., a self-adaptive selection of Harris corners and a multiple-constraint corner matching. (i) We utilize Harris corners, obtained with low computational complexity and high robustness, as features to align images. We fragment each original image into several regions and select corners within each region according to the normalized variance of region grayscales. Such a self-adaptive selection guarantees that corners are distributed such that more will be selected from regions with richer texture information. In addition, our algorithm avoids the possible clustering of corners. (ii) Upon filtering out a large number of inappropriate corners according to their position information, we generate an initial set of matching-corner pairs based on grayscales of adjacent regions around each corner. Finally we apply multiple constraints on every two candidate pairs, e.g., their midpoints, distances, and slopes, to remove incorrectly matched pairs. As such, we are able to significantly reduce the number of iterations needed in traditional Random Sample Consensus (RANSAC) algorithm [12]. The video panoramic image stitching can then be performed in a more efficient manner. Moreover, our algorithm is robust to errors caused by the camera shake because optimal parameter values will be obtained in a self-adaptive manner during the image stitching.

The rest of this paper is organized as: Section “Related Work” briefly discusses related work; Section “Materials and Methods” introduces in detail our methodology; Section “Results and Discussion” describes experimental results; Section “Limitations of the Study, Open Questions, and Future Work” discusses some open questions along with future research directions; and finally Section “Conclusions” concludes.

## Related Work

Reddy and Chatterji [3] discussed an extension of the well-known phase correlation technique to cover translation, rotation, and scaling during the image matching. Fourier scaling properties and Fourier rotational properties were used to find scale and rotational movement, and the phase correlation technique determined the translational movement. The algorithm presented in this work is characterized by its insensitivity to translation, rotation, scale, and noise as well as by its low computational cost. The authors believed that their matching algorithm was exact except for scaling where it was found to deviate in the third place of decimal probably due to the nonlinear processing.

Since to solve the problem of modeling and analyzing pushbroom sensors commonly used in satellite imagery is difficult and computationally intensive, the authors presented a simplified model of a pushbroom sensor (the linear pushbroom model) in [1]. Their model has the advantage of computational simplicity while at the same time giving very accurate results compared with the full orbiting pushbroom model. Besides remote sensing, the linear pushbroom model is also useful in many other imaging applications, and this model leads to theoretical insights that are approximately valid for the full model as well.

Consistency of image edge filtering is of prime importance for 3D interpretation of image sequences using feature tracking algorithms. To cater for image regions containing texture and isolated features, the authors in [4] presented a combined corner and edge detector based on the local auto-correlation function. They successfully demonstrated that their proposed detector is able to perform with good consistency on natural imagery.

Lacey, Pinitkarn, and Thacker [12] compared the use of RANSAC for the determination of epipolar geometry for calibrated stereo reconstruction of 3D data with more conventional optimisation schemes. The authors illustrated the poor convergence efficiency of RANSAC and highlighted the need for an a-priori estimate of outlier contamination proportion. An algorithm was suggested to make better use of the solutions found during the RANSAC search while giving a convergence criteria that is independent of outlier proportion.

A review of recent and classic image registration methods was presented in [13]. Approaches reviewed were classified according to their nature (area-based and feature-based) and four basic steps of the image registration procedure: feature detection, feature matching, mapping function design, and image transformation and resampling. The authors also discussed problematic issues of image registration and provided an outlook for the future research.

Sivic and Zisserman [5] described an approach to object and scene retrieval that searches for and localizes all occurrences of a user outlined object in a video. The object was represented by a set of viewpoint invariant region descriptors so that recognition can proceed successfully despite changes in viewpoint, illumination, and partial occlusion. The authors used the temporal continuity of the video to track regions to reject unstable regions and reduce the noise effect in the descriptors.

The performance of descriptors computed for local interest regions was compared in [6]. Mikolajczyk and Schmid believed that descriptors should be distinctive and at the same time robust to changes in viewing conditions as well as to errors of the detector. Their evaluation used as criterion recall with respect to precision and was carried out for different image transformations. In addition, they also proposed an extension of the SIFT descriptor and showed that it outperforms the original method.

A method for image similarity measure was presented in [2], where a hand-drawn rough black-and-white sketch was compared with an existing database of full color images (art works and photographs). The system created the evaluation of nonprecise, easy-to-input sketched information and can then provide the user with options of either retrieving similar images in the database or ranking the quality of the sketch against a given standard. Alternatively, the inherent pattern-matching capability of the system can be utilized to allow detection of distortion in any given real time-image sequences in vision-driven ambient intelligence applications.

Nister, Naroditsky, and Bergen [7] presented a system to estimate the motion of a stereo head or a single moving camera based on video input. The system operates in real-time with low delay and the motion estimates were used for navigational purposes. Point features were matched between pairs of frames and linked into image trajectories at video rate. Robust estimates of the camera motion were then produced from the feature tracks using a geometric hypothesize-and-test architecture. The authors successfully applied the pose estimation method to video from aerial, automotive, and handheld platforms.

Zhu et al. [8] introduced the concept of distribution quality to quantify the distribution of seed points for triangle constrained image-matching propagation. An intensive experimental analysis was illustrated using two different stereo aerial images and, based on the experimental results, a seed point selection strategy was proposed. An automatic selection method was then introduced to provide good distribution quality for a defined number of seed points. Seed points with proper distribution are able to provide better matching results and the distribution quality is a useful descriptor to the distribution of seed points.

Zhao et al. [9] presented a regional corner-selection algorithm to overcome the disadvantage found in traditional Harris detector, i.e., corners tend to cluster around regions with richer texture information. They first fragmented the original image into several regions, then selected a fix number of corners in each region and ultimately applied a scaling parameter to finalize the corner selection. Their algorithm is able to select corners with relatively high quality but there exist some issues. We will further discuss this algorithm in later sections because it is closely related to our proposed algorithm in this paper.

In [14], the authors defined digital image mosaics as image registration and fusion of many overlapping images or multiple video frames into a single wide field of view image or dynamic panoramic image. They introduced four basic types of image mosaics based on the characters of images to be mosaic. The state-of-the-art image mosaics research was summarized and new research directions were discussed.

The authors in [10] presented a fast image mosaic algorithm based on feature points matching. They proposed two algorithms. The first one is to import a new filtering method for matching points by choosing pairs of correlated feature points with a clustering algorithm aiming at the disadvantage of the RANSAC algorithm. The second algorithm presents a new method of blending images by using optimum path best-matched-line combined with pixel brightness weighting function in HSI color space. Their methodologies were able to remove the gusting phenomenon and result in a brightness blending image, and, in a more efficient manner.

Cao et al. [11] proposed to extract features from edges of an image so that the multi-scale image fusion and mosaicing can be performed. An edge-smoothing pyramid was built and stable features of image registration were extracted. They then reused the multi-scale representation to fuse registered images and thus eliminated the cost of mosaicing. Results indicated that their algorithm can eliminate false feature matches, enhance the image transformation precision, and reduce the computation cost of registration and following mosaic.

## Materials and Methods

### Improved Harris Corner Selection

The Harris algorithm [4] detects corners by calculating the intensity change of a pixel after it is shifted in some direction. According to [4], for a given pixel 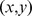, an autocorrelation function of the surrounding area of 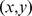, 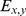, is defined in Eq. (1), where 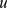 and 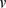 are small shifts in 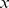 and 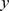 coordinates, respectively; and 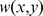 is the Gaussian function to filter out noise.
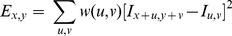
(1)


The Taylor expansion of 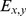 is shown in Eq. (2), and 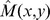 is the autocorrelation matrix (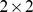 and symmetric), calculated as in Eq. (3), where 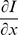 and 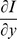 are partial derivatives of the pixel in 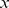 and 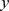 coordinates, respectively; 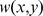 is the Gaussian function to filter out noise; and 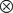 is the convolution operation.
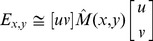
(2)

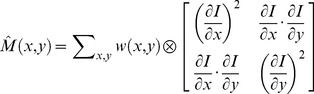
(3)


The autocorrelation matrix, 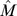, can be used to detect corners. To be more specific, Eq. (4) defines a response function for the matrix, where 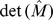 and tr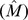 are the determinant and trace of 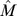, respectively; and 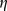 is a parameter in the range of [0.04, 0.06]. If the *R* value of a pixel is greater than a threshold, such a pixel will be selected as a Harris corner. Note that *R* depends on various characteristics of actual images, size and texture for example. As a result, *R* is usually determined indirectly: pixels are sorted in a descending order of their *R* values; then the first Sum (a predefined total number, see greater details in Eq. (8)) pixels are selected as corners. Notice that the discovery of meaningful corners does not depend on region boundaries because the calculation of *R* value is not tied with fragmented regions.
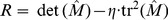
(4)


Harris detector only involves the first-order difference and filtering operations of pixel grayscale, with low computational complexity. A large number of corners will be detected in regions with rich texture, whereas fewer corners will be selected in regions with less texture information. Therefore, selected corners are not evenly distributed. In other words, corners tend to cluster around regions with richer texture. Zhao et al. [12] proposed a regional corner-selection algorithm, where they fragmented the original image into several regions. A fix number of corners with top *R* values were selected in each region as candidate corners, and all such candidate corners (a total of Sum) were then sorted in their *R* values. Finally a scaling parameter, 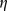, whose range is (0, 1), was applied to finalize the corner selection, i.e., a total of 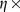 Sum corners will be generated. To assure that each region contains some finalized corners, they iteratively applied different 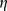 values in an ascending order and the iteration was terminated as soon as there was at least one finalized corner for each region. This algorithm is able to select corners with relatively high quality but some issues exist. (i) When corners are evenly distributed the iteration will be terminated early with a small 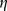 value, which results in fewer corners than necessary and will affect the corner matching thereafter. (ii) Corners selected in regions with rich texture contain redundant, neighboring corners. (iii) The value of 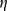 is biased to outlier regions, i.e., regions with extremely rich or poor texture.

We believe that the corner selection should be proportional to region texture information and, at the same time, should avoid the possible clustering as much as we can. To handle such a tradeoff, we propose to select corners in each region according to the normalized variance of region grayscales. Our corner selection is self-adaptive and decomposed into several steps.

**Figure 1:**
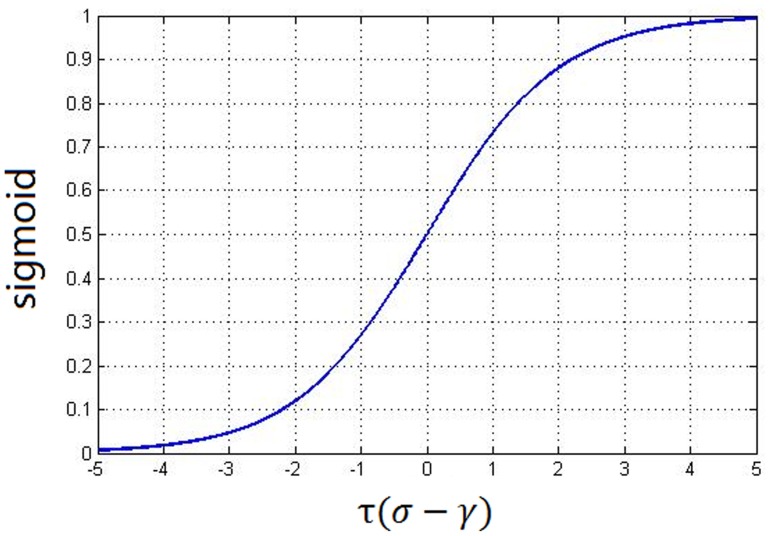
The Sigmoid function, where it is clearly demonstrated that the critical value range of 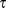 is [−5, 5].



- We fragment the original image into 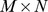 regions. For each region, a texture weight, 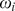, is calculated according to the grayscale variance of that region. To be more specific, such a weight should be able to offset the region texture feature. In other words, the larger the grayscale variance in a region, the more influence on the weight of that region. Due to its characteristics of smoothness and progressiveness (Figure 1), we utilize the Sigmoid function (demonstrated in Eq. (5), where 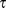 is a ratio parameter; 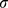 is the variance; and 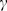 is the offset amount) to normalize the grayscale variance in a region (demonstrated in Eq. (6), where *i* is an integer in the range of (1, 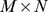); 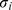 is the grayscale variance of the 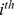 region; and 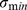, 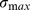, and 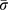 are the minimum, maximum, and expected values of the grayscale variance, respectively). After we calculate the normalized variance of each region, 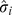, it is in turn used to determine the weight for that region (Eq. (7)). Finally we use 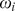 to determine the number of corners, 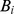, in each region (Eq. (8)), where Sum is the predefined total number of corners for all regions.
- *R* value is calculated for each pixel in each region according to Eq. (4). Corners with maximum or minimum *R* values are eliminated.
- All pixels in each region are sorted in a descending order of their *R* values. The first 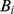 pixels in each region are then selected as corners.

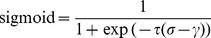
(5)

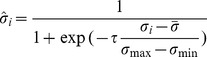
(6)

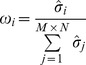
(7)

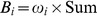
(8)


Note that in Eq. (6) we set the value of  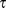 to 10 because it is clearly demonstrated in Figure 1 that the critical variable range is the grayscale variance (i.e., the mean variance) as the offset amount because compared with the median, the mean can better offset outliner regions.

### Multiple-Constraint Corner Matching

The traditional RANSAC algorithm is inefficient, especially when stitching a large number of images and when these images have similar features. It thus does not meet the real-time requirement commonly found in video panorama stitching. Note that in the field of video panorama stitching, more often than not, adjacent images have highly similar features with each other. Based on this insight, we propose to apply multiple constraints on candidate matching-corner pairs to remove incorrectly matched pairs. As such, we can significantly reduce the number of iterations needed in traditional RANSAC algorithm.

- Step 1: Corner similarity matrix between adjacent images. Suppose that there are two images to be aligned, 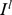 on the left hand side and 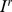 on the right hand side, both of which have the same resolution of 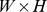. One corner from the left image is denoted 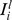 (with coordinates 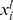 and 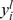, and the intensity of 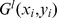) and another corner from the right image is denoted 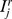 (with coordinates 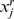 and 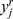, and the intensity of 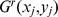). These two corners, 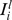 and 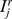, can be aligned with each other if the following constraints are satisfied: (i) the 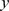 coordinate difference of these two corners is less than a threshold; (ii) the 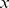 coordinate of the left corner is no less than that of the right corner; (iii) the difference of *R* values of these two corners is less than a threshold; and (iv) there is a high intensity correlation between these two corners. The first three constraints are formalized in Eq. (9), where 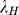 and 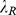 are thresholds in constraints 1 and 3, respectively. Note that constraint 2 requires knowing in advance which image is the left one and which is the right one. In other words, our algorithm is only working on camera panoramas where the panning direction (left or right) is known in advance and does not change. Fortunately, such a restriction is not difficult to be satisfied in real-world applications. As for the last constraint, we need to make use of the normalized cross-correlation (NCC) function described in [14]. Suppose that the similarity window size is 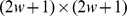, NCC is calculated as in Eq. (10), where 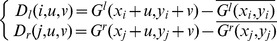, and 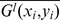 and 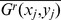 are the mean intensity of windows around corners 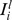 and 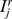, respectively. Finally, we utilize Eq. (11) to calculate pairwise corner similarity and create a similarity matrix between adjacent images 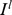 and 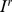.

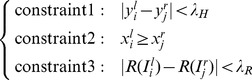
(9)

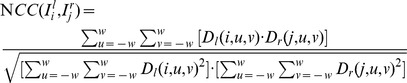
 (10)
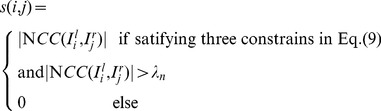
(11)

- Step 2: Initial set of matching-corner pairs. A set of indexes of matching-corner pairs is generated by the following procedure: in each row of the similarity matrix obtained in Step 1, we find the column index such that the corresponding cell in the matrix has the maximum value for that row, and the pair of (row index, column index) is added into the set. After we process all rows in the matrix, we will obtain a set 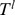. Such a procedure is formally described in Eq. (12), where 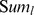 is the predefined total number of corners in the left image 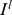.

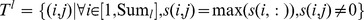
(12)Similarly, we can obtain another set 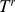 by searching the maximum row index for each column. Eq. (13) is the formal description of this procedure where 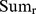 is the predefined total number of corners in the right image 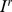.
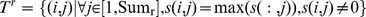
(13)


In general, 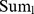 in Eq. (12) and 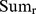 in Eq. (13) can take different values. In our algorithm we use the same value for these two parameters. Now we compare two sets, 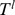 and 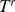. If a row index and a column index happen to have each other as the other component in a pair, their similarity should be adjusted. In other words, if two corners mutually find their “best” match as each other, such a pair will have an adjusted similarity value of 1. Eq. (14) formalizes this procedure of similarity adjustment. Note that this idea is also known as the “stable marriage” criteria, and was proposed in literature, [7] for example. Our methodology is different from existing methodologies in that (i) we do not discard pairs not satisfying the stable marriage criteria and (ii) the calculation of the similarity measurement is only one of the steps in our algorithm.
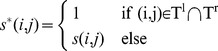
(14)


Finally we generate an initial set of matching-corner pairs *T* by a union of 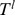 and 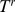 (Eq. (15)). Note that this initial set of pairs is already reduced in size compared with NCC rough match in traditional RANSAC algorithm because as exhibited in Eq. (9) we have already filtered out some inappropriate corners according to their coordinate values in respective regions.
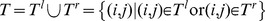
(15)

**Figure 2:**
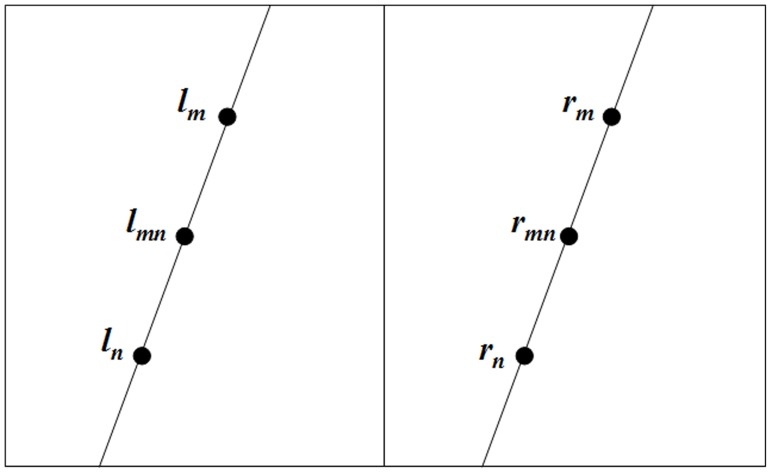
Two initially matching-corner pairs, 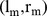 and 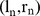, along with their respective midpoints: 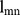 between 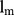 and 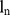 and 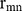 between 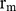 and 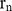.



- Step 3: Multiple constraints on matching pairs. Considering two initial matching-corner pairs in Figure 2, 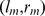 and 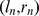, along with their respective midpoints, i.e., 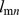 between 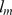 and 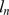 and 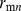 between 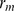 and 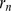. Let 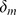 and 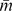 be the slope and length of the segment formed by 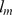 and 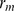, respectively; and 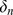 and 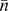 be the slope and length of the segment formed by 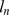 and 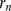, respectively. We design multiple constraints to be applied to these two matching-corner pairs, as shown in Eq. (16).

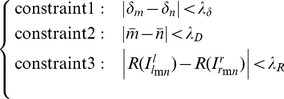
(16)The intuition of Eq. (16) is that, between two matching pairs, not only the R values (Eq. (4)) of their respective midpoints should be correlated (constraint 3), but also the slope (constraint 1) and length (constraint 2) of the segments formed between these two pairs should be similar with each other as well. According to multiple constraints specified in Eq. (16), we calculate pairwise similarity between every two initial matching pairs using Eq. (17), and generate a matrix *D* of size 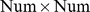, with Num being the cardinality of *T*.
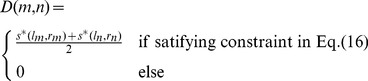
(17)

- Step 4: Final, reduced set of matching-corner pairs. Among all initial matching-corner pairs, according to Eq. (18), we search for a special pair, *t*, which has the strongest correlation with all other pairs.

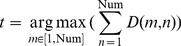
(18)We then refer back to the matrix *D* generated in Step 3, and find all initial matching pairs that have non-zero correlation with the aforementioned special pair, *t*. In other words, an initial matching-corner pair will be output to the final, further reduced set as long as a non-zero value is found at the cell in *D* corresponding to this pair and the special pair *t*. Eq. (19) formally specifies this final selection step, and the resultant set 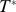 is the finalized, reduced set of matching-corner pairs. Note that the size of 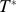 is further reduced from that of *T*, and we explained earlier in Step 2 that *T* is already reduced in size compared with NCC rough match in traditional RANSAC algorithm.
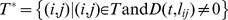
(19)


Steps 1, 3, and 4 reflect our proposed heuristics with regard to the spatial consistency. Similar heuristics were presented in literature (for example [5]). It is true that heuristics described in [5] are in a more general formulation. However, our more specific formulation is exactly our unique contribution: as mentioned earlier in Section “Introduction”, our algorithm is motivated by two insights on adjacent images, i.e., their small overlapping and their small difference in translation, rotation, and scaling. In other words, Eq. (9) and Eq. (16) fit better the video panoramic image stitching scenario because we take into account unique features of adjacent images to be stitched. Consequently, our methodology is more efficient (see Section “Results and Discussion” for greater details).

### Image Stitching

After we obtain a finalized set of matching-corner pairs between two original images, we select one image as the reference image (the first image) and the other as the image to be aligned (the second image). We calculate the affine transformation parameters using the RANSAC algorithm. Suppose that 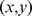 is a pixel in the reference image and 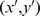 is the corresponding pixel in the other image. The relationships between their coordinates are illustrated in both Eq. (20) and Eq. (21), where 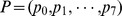 is the vector of affine transformation parameters, which can be easily calculated from 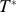. Based on the values of these parameters we then map pixel coordinates in the second image into the coordinate system of the reference image. In addition, the light conditions may vary among different cameras; therefore, the panorama to be generated may be inconsistent in its overall intensity. To obtain a smooth transition in overlapping areas between images to be stitched, we utilize the weighted-sum method introduced in [14] to perform a gradual fading-in and fading-out image-stitching process to generate the final video panoramic image. Suppose that 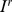 is the reference image and 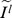 is the transformed source image calculated based on *P*, the stitched image, 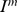, is generated according to Eq. (22), where 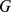 stands for pixel intensity; 
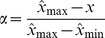
, 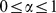, and 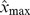 and 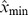 are maximum and minimum coordinates in the overlapping region, respectively.
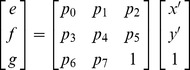
(20)

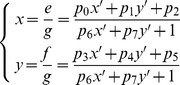
(21)

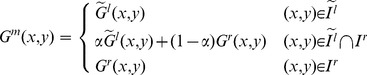
(22)


During the alignment and stitching of the first 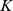 frames, an initial set of optimal transformation parameters, 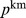, can be obtained according to Eq. (23), where 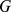 stands for pixel intensity. Then, newly generated, real-time frames will reuse these optimal parameters for the stitching process. This strategy is appropriate for smooth (non-shaking) panorama generation as it can avoid unnecessary feature detection/matching. When there does exist a shaking camera, since there are more random alignments between frames, initially optimal parameters may not work for new frames. In this scenario, our solution is to calculate the similarity between overlapping regions of two images. When the similarity is lower than a predefined threshold, a new set of optimal parameters can be automatically re-calculated and applied to newly generated frames. As such, our strategy is a self-adaptive one and can well handle errors resulted from the camera shaking. In addition, the similarity between overlapping regions is regarded as a by-product of the stitching process defined in Eq. (22). As a result, only nominal time is needed for calculating the aforementioned similarity, and the algorithm efficiency is thus not much affected.
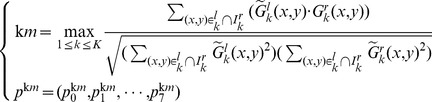
(23)


## Results and Discussion

### Experimental Environment and Parameter Setup

Note that all data and images generated from our experiments, unless stated otherwise, are publicly available. No restrictions are placed on producing derivatives of original data or images except that formal acknowledgement to original inventors is required. Examples of such acknowledgement include but not limited to publication citations. The intellectual property belongs to Fuzhou University.

We used a PC (CPU E2200+2.2 GHz, 4 GB Memory, Matlab 7.0) to conduct our experiments, and the image resolution was set to 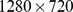.

According to our previous experience, we set experimental parameters as follows:

- The 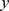 coordinate difference of adjacent cameras was no greater than one third of the image height, i.e., 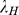 in Eq. (9) was set to 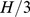;

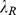
 in Eq. (9) was set to 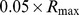;
- The horizontal overlapping was no great than 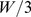;
- The similarity window size in Eq. (10) was set to 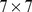, i.e., 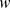 was set to 3;
- The similarity threshold 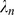 in Eq. (11) was set to 0.75;

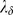
 in Eq. (16) was set to 0.5;
- The original image was segmented into regions of size 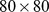, and the number of corners for each region was set to six, therefore, 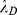 in Eq. (16) was set to 14 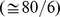; and

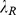
 in Eq. (16) was set to 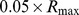.

### Evaluation on Corner Selection

**Figure 3:**
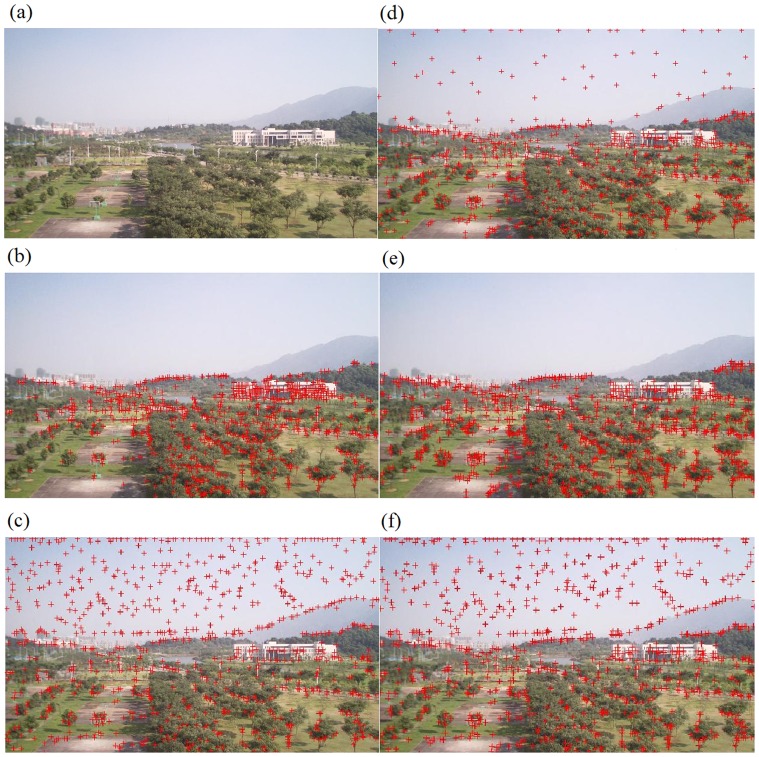
Experiments on corner selection: (a) Original image; (b) Harris detector; (c) The regional corner-selection algorithm; (d) Our algorithm with 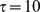; (e) Our algorithm with 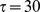; (f) Our algorithm with 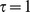.

The experimental results are demonstrated in Figure 3. The corner-selection result from the traditional Harris detector is shown in Figure 3(b). Because the original image, Figure 3(a), was not fragmented at all, corners tended to cluster around regions with richer texture. The result from the regional corner-selection algorithm in [12] is demonstrated in Figure 3(c), where corners were evenly distributed without taking into account the texture difference among various regions. On the contrary, our algorithm (Figure 3(d)) handled well the tradeoff discussed earlier in Subsection “Improved Harris Corner Selection” (i.e., the number of corners should be proportional to region texture information and, at the same time, the corner clustering should be avoided as much as possible). Also note that different values of 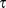 in Eq. (6) may significantly affect the corner-selection result. To be more specific, with the increasing value of 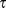, Figure 3(e) for example, the texture difference will be amplified and the result will be closer to that of The Harris Detector. On the other hand, if the value of 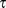 is small, Figure 3(f) for example, the texture difference will be diminished and the result will be closer to that of the regional corner-selection algorithm in [9]. By choosing different values of 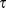, our corner-selection algorithm is flexible in terms of serving various purposes from users€ viewpoint.

### Evaluation on Corner Matching Efficiency

**Figure 4:**
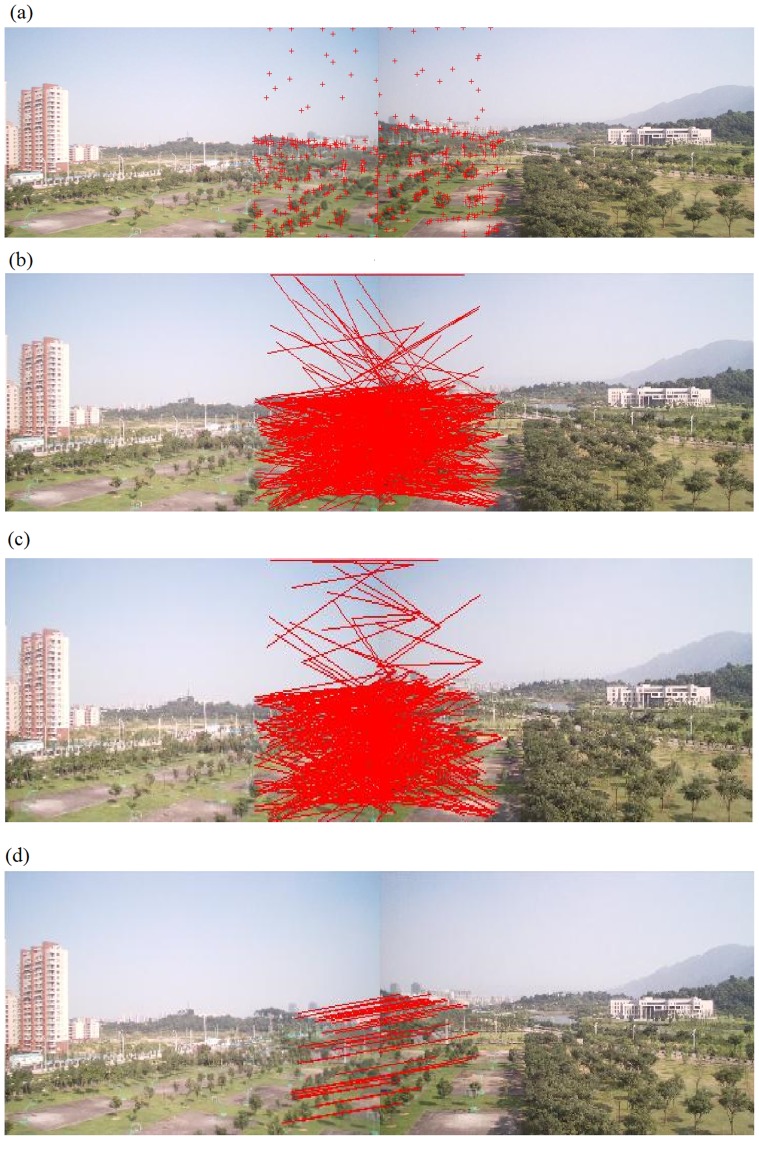
The first experiment on the corner matching efficiency: (a) Selected corners (using our algorithm) from two original images; (b) Corner matching by traditional RANSAC algorithm (NCC rough match); (c) Initial set of matching-corner pairs in our algorithm; (d) Final set of matching-corner pairs in our algorithm.

**Figure 5:**
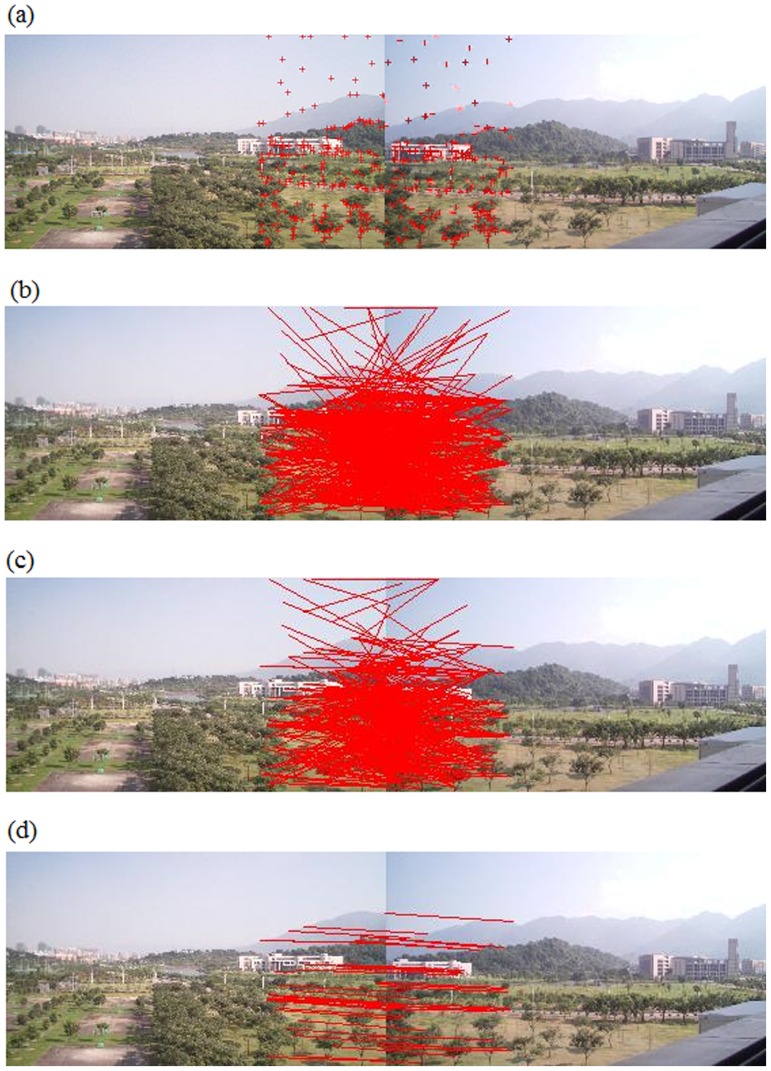
The second experiment on the corner matching efficiency: (a) Selected corners (using our algorithm) from two original images; (b) Corner matching by traditional RANSAC algorithm (NCC rough match); (c) Initial set of matching-corner pairs in our algorithm; (d) Final set of matching-corner pairs in our algorithm.

Experimental results in Subsection “Evaluation on Corner Selection” have clearly indicated that our corner-selection methodology is superior to the methodology in the traditional Harris detector. Therefore, experiments in this section will be based upon our corner-selection algorithm, so that a fair comparison can be performed between different corner-matching processes. Consequently, experiments in this section do not have all possible four combinations between corner selection and corner matching methodologies. Our experimental results are demonstrated in Figures 4, 5 and Tables 1, 2, and 3. Figure 4(a) contains two original images with corners selected using our algorithm. We chose the right one-third region of the left image and the left one-third region of the right image as two regions to perform corner matching. So we had a total of 45 segmented regions 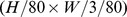, and the total number of corners is 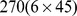. The total number of matching-corner pairs from NCC rough match in the traditional RANSAC algorithm was 528 (Figure 4(b)), and the total numbers of initial and finalized matching-corner pairs from our algorithm were 456 (Figure 4(c)) and 41 (Figure 4(d)), respectively. For the purpose of analysing experimental results in a clearer manner, we further summarize these results in Table 1: the traditional RANSAC algorithm needs to calculate the extremely time-consuming NCC function for all pairwise combinations of corners (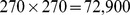 in this example), and our algorithm only need to consider a small number of combinations (17,993 in this example, which is less than 25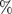 of 72,900). As a result, our multiple-constraint corner matching was almost four times faster than NCC rough match in the traditional RANSAC algorithm (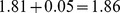 seconds vs. 7.20 seconds). In addition, Figure 4(d) clearly demonstrates that most of the matching pairs were correct ones. Figure 5 and Table 2 demonstrate similar results on another experiment. Due to the limited space, more experimental results can be found at: http://www.soc.southalabama.edu/huang/ImageStitching/ExperimentResults.rar. Overall (average and variation) quantitative results for these additional experimental results are summarized in Table 3.

**Table 1:** Efficiency comparison between traditional RANSAC and our algorithm (I).

	Total number of corner pairsto be processed	Total number of generatedcorner pairs	Time spent (second)
NCC rough match intraditional RANSAC	72,900( = 270×270)	528	7.20
Our algorithm (set *T*)	17,993	456	1.81
Our algorithm (set T*)	**456**	**41**	**0.05**

**Table 2:** Efficiency comparison between traditional RANSAC and our algorithm (II).

	Total number of corner pairsto be processed	Total number of generatedcorner pairs	Time spent (second)
NCC rough match intraditional RANSAC	72,900( = 270×270)	534	6.80
Our algorithm (set *T*)	16,176	453	1.63
Our algorithm (set T*)	**453**	**45**	**0.08**

**Table 3:** Efficiency comparison between traditional RANSAC and our algorithm (Overall).

	Total number of corner pairs to be processed	Total number of generated corner pairs	Time spent (second)
	Average/Variation	Average/Variation	Average/Variation
NCC rough match intraditional RANSAC	72,900/0	532/16	7/0.07
Our algorithm (set *T*)	17,087/37	455/4.5	1.73/0.02
Our algorithm (set T*)	**455/4.5**	**43/6**	**0.07/0.003**

#### Evaluation on image stitching

We conducted two different sets of experiments to evaluate the image-stitching result. Notice that all final panoramic images in this section were obtained using the methodology discussed in Subsection “Image Stitching”.

#### The first set of experiments on stitching evaluation

**Figure 6:**
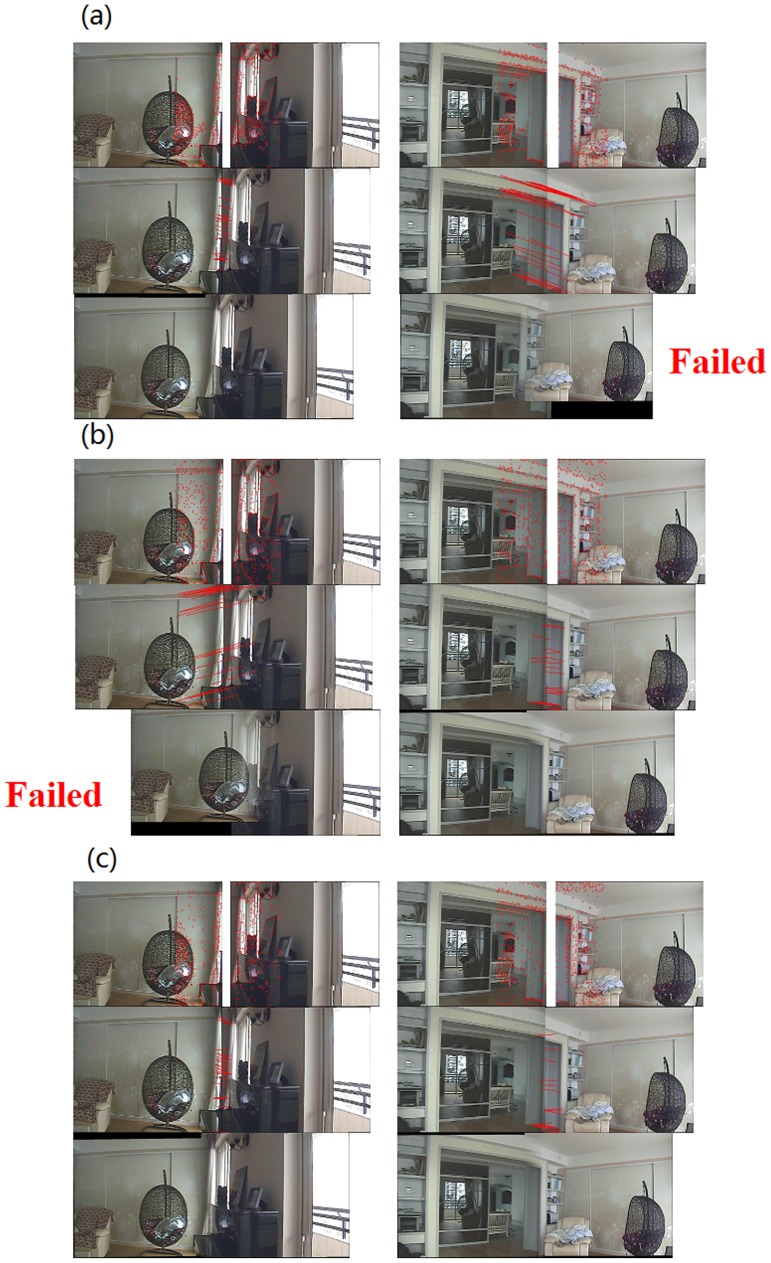
Comparison of stitching results from three different algorithms (I): (a) Traditional Harris detector; (b) The regional corner-selection algorithm in [9]; (c) Our presented algorithm.

**Figure 7:**
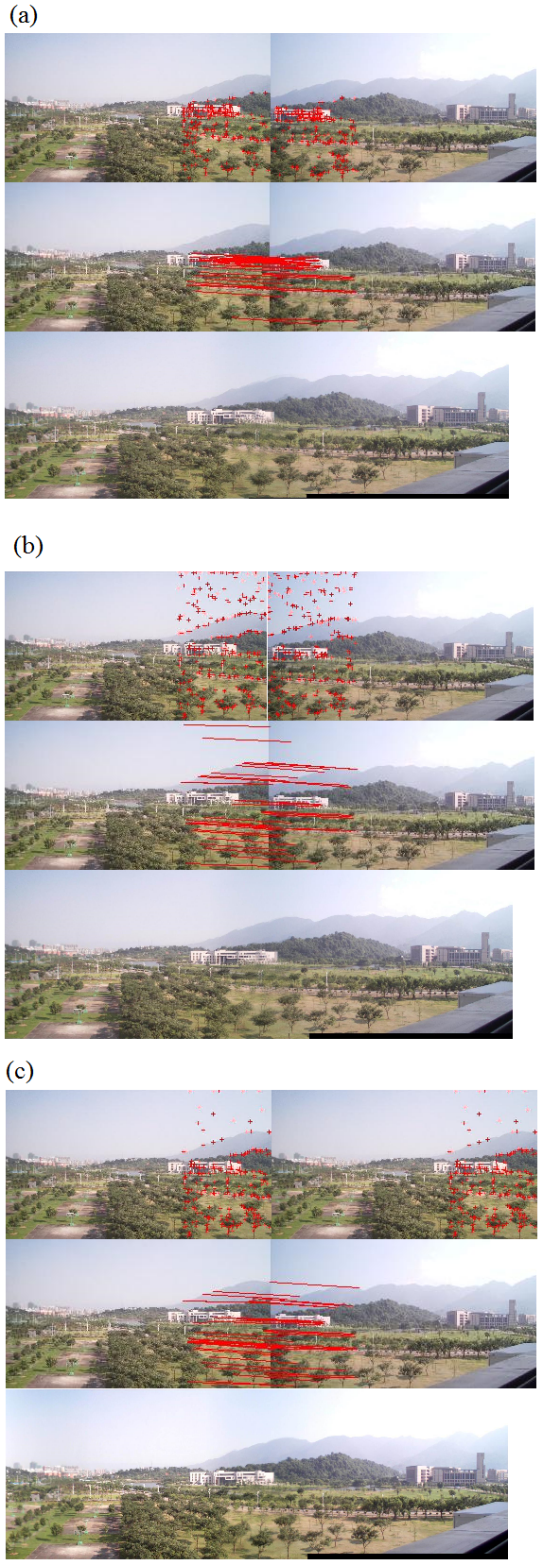
Comparison of stitching results from three different algorithms (II): (a) Traditional Harris detector; (b) The regional corner-selection algorithm in [9]; (c) Our presented algorithm.

The first set of experiments was performed to compare stitching results from the traditional Harris detector, the regional corner-selection algorithm in [9], and our presented algorithm, respectively. Experimental results are demonstrated in Figures 6, 7 and Tables 4, 5. Let us first discuss results in Figure 6 and Table 4.

**Table 4:** Qualitative and quantitative analysis on three different algorithms (I).

	Left Part		Right Part	
Algorithm	Successful stitching ornot? (qualitative)	Similarity betweenneighboring regions to bestitched (quantitative)	Successful stitchingor not? (qualitative)	Similarity between neighboring regions to be stitched (quantitative)
A (Traditional Harris detector)	Yes	0.9479	No	0.8613
B (Regional corner-selection algorithm)	No	0.8088	Yes	0.9827
C (Our presented algorithm)	Yes	0.9442	Yes	0.9788

**Table 5:** Qualitative and quantitative amalysis on three different algorithms (II).

Algorithm	Successful stitching or not?	Similarity between neighboring regions to be stitched
A (Traditional Harris detector)	Yes	0.9737
B (Regional corner-selection algorithm)	Yes	0.9735
C (Our presented algorithm)	Yes	**0.9842**

In Figure 6, there are two pairs of images to be stitched. Due to the rich texture information contained in the original images, the traditional Harris detector was able to obtain correct matching corners between two images on the left pair and was successful in stitching these two images. However, the traditional Harris detector was not successful in stitching images on the right pair due to not enough texture information contained in original images. The regional corner-selection algorithm had exactly opposite results: successful in stitching images on the right pair but not successful on the left pair. The example in Figure 6 may suggest that, the traditional Harris detector and the regional corner-selection algorithm are appropriate for scenarios of rich texture and for scenarios without enough texture information, respectively, but for not both. Consider the example demonstrated in Figure 6, the left pair of images has small overlapping regions but rich texture information. On the contrary, the right pair of images has large overlapping regions but not enough texture information. On one hand, the traditional Harris detector was able to handle the left pair of images successfully but failed to deal with the right pair (Figure 6(a)). On the other hand, the regional corner-selection algorithm was able to handle the right pair but failed on the left pair (Figure 6(b)). As a comparison, our algorithm successfully handled both the left and right pairs of images because it handles well the tradeoff discussed earlier (i.e., the number of corners should be proportional to region texture information and, at the same time, the corner clustering should be avoided as much as possible). Furthermore, as shown in Table 4, a quantitative analysis indicates that our algorithm was able to obtain high intensity similarity between neighboring regions to be stitched. Therefore, the observation from Figure 6 and Table 4 is that, our algorithm is appropriate for various scenarios (rich texture information or not) and is able to obtain satisfactory intensity similarity between original images.

Figure 7 demonstrates stitching results for another pair of images. Compared with the two pairs of images in Figure 6, original images in Figure 7 contain more evenly distributed texture information. As a result, all three algorithms were able to obtain a successful stitching. However, as shown in Table 5, our algorithm is superior to the other two algorithms in terms of the quantitative analysis on the intensity similarity between original images.

#### The second set of experiments on stitching evaluation

**Figure 8:**
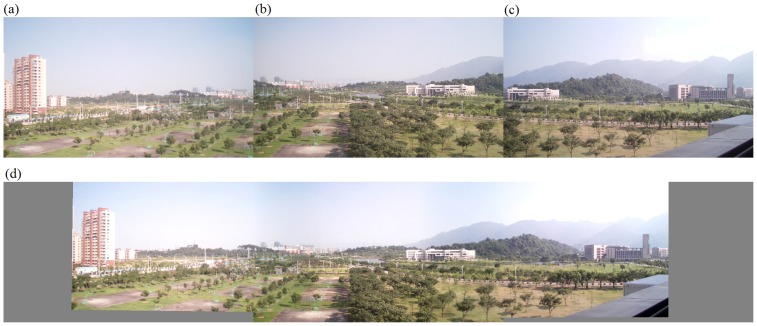
Experiments on the entire image-stitching algorithm (I): (a) Video image 1; (b) Video image 2 (reference image); (c) Video image 3; (d) Generated video panoramic image.

**Figure 9:**
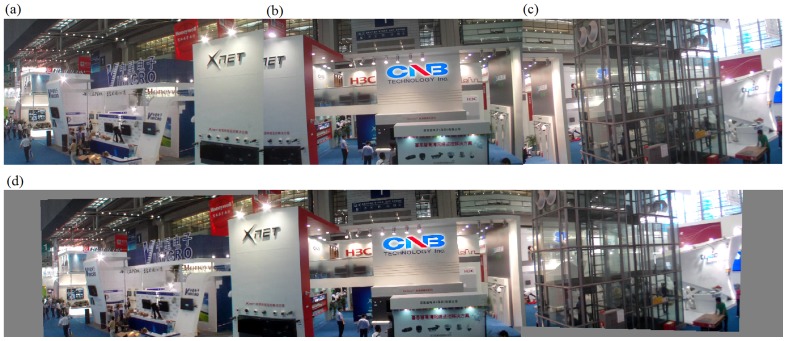
Experiments on the entire image-stitching algorithm (II): (a) Video image 1; (b) Video image 2 (reference image); (c) Video image 3; (d) Generated video panoramic image.

**Figure 10:**
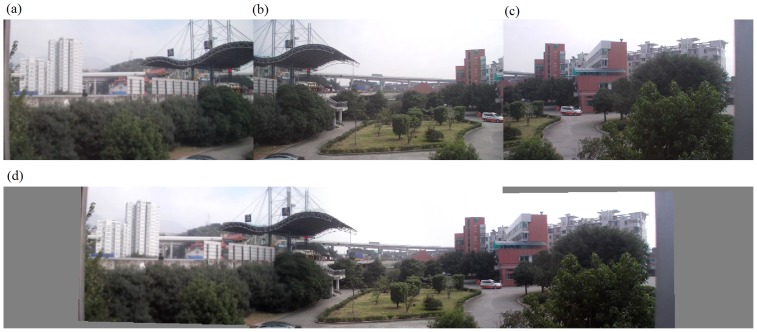
Experiments on the entire image-stitching algorithm (III): (a) Video image 1; (b) Video image 2 (reference image); (c) Video image 3; (d) Generated video panoramic image.

**Figure 11:**
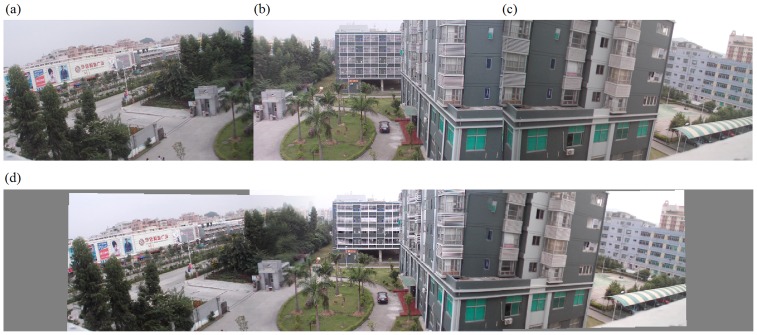
Experiments on the entire image-stitching algorithm (IV): (a) Video image 1; (b) Video image 2 (reference image); (c) Video image 3; (d) Generated video panoramic image.

**Figure 12:**
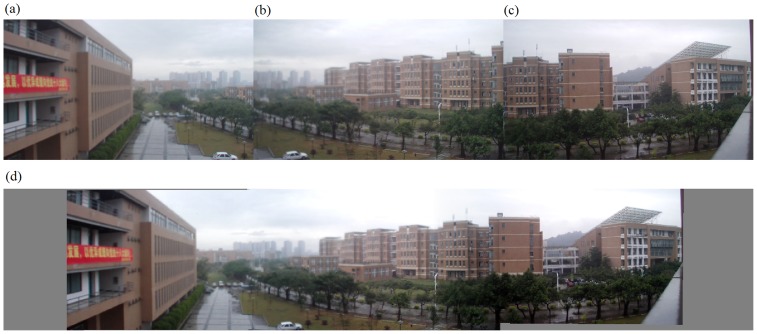
Experiments on the entire image-stitching algorithm (V): (a) Video image 1; (b) Video image 2 (reference image); (c) Video image 3; (d) Generated video panoramic image.

The second set of experiments was performed on our algorithm alone. In fact, we implemented the entire algorithm (from corner selection to corner matching and finally to image stitching) in Visual C++2005. Results are demonstrated in Figures 8 to 12. Notice that all original images, i.e., (a), (b), and (c) in Figures 8 to 12 have small differences in translation, rotation, and scaling between each other. As discussed earlier in Section “Introduction”, one motivation of our algorithm is the small difference in translation, rotation, and scaling on adjacent images to be stitched. Figure 8 is explained in detail here. We performed our proposed Harris corner selection and multiple-constraint corner matching between Figure 8(a) and Figure 8(b), and between Figure 8(b) and Figure 8(c), respectively. After we obtained two finalized sets of matching-corner pairs, we selected Figure 8(b) as the reference image and calculated the projective transformation parameters. We then mapped pixel coordinates in Figure 8(a) and Figure 8(c) into the coordinate system of Figure 8(b), respectively. Finally we performed a gradual fading-in and fading-out image-stitching process. The final result in Figure 8(d) clearly demonstrates that (i) our corner matching was accurate; (ii) we obtained a smooth transition in overlapping areas between images to be stitched; and (iii) the panorama generated satisfied human visual requirements. In addition, the preview speed of the generated panorama was around 20 frames per second, which satisfied the real-time requirement commonly found in video panorama stitching. Figures 9 to 12 exhibited convincing results from additional images. Likewise, due to the limited space in this paper, more results have been stored at the following location and can be freely downloaded: http://www.soc.southalabama.edu/~huang/ImageStitching/ExperimentResults.rar. Notice that as discussed earlier in Subsection “Image Stitching”, our self-adaptive strategy to obtain optimal parameter values during the image stitching can well handle errors resulted from the camera shake. Successful stitching results demonstrated in a video clip (uploaded to aforementioned extra experimental results) can justify our claim.

## Limitations of the Study, Open Questions, and Future Work

1. There are a number of scenarios where our proposed methodology might be affected. In our future work, it will be worthwhile to explore how we effectively deal with such challenging scenarios. Some example cases are listed as follows.
  - What happens in case there are a lot of movements in the frame? For example, when taking videos in cars or other vehicles.
  - Besides the camera shake, can other fast movements be well handled? If so, how much overlapping between adjacent frames is considered adequate?
  - How to handle changes in lighting conditions? For example, when nearby cameras use a flash.
2. To better satisfy the real-time requirement commonly found in video panorama stitching, there is a tradeoff between obtaining more features for matching within a frame and simply capturing more frames to allow for greater overlapping. How to effectively handle such a tradeoff can be another interesting future work.
3. Other future research directions include, but are not limited to, (i) the automatic determination of the total number of corners according to the image texture information and (ii) the motion ghost challenge during the image stitching.

## Conclusions

We presented an innovative algorithm to handle challenges in video panoramic image stitching, e.g., small overlapping regions, extremely time-consuming stitching processes. Our contribution can be summarized as (i) an improved, self-adaptive selection of Harris corners and (ii) a multiple-constraint corner matching. To better select high-quality corners, we fragmented original images into numerous regions and then selected corners within each region based on the normalized variance of region grayscales. This self-adaptive selection assured that corners are evenly distributed (i.e., proportional to region texture information) and, at the same time, avoided the corner clustering as much as possible. To overcome the inefficient corner matching in the traditional RANSAC algorithm, we first filtered out a large number of inappropriate corners according to their position information. We then generated an initial set of matching-corner pairs based on grayscales of adjacent regions around each corner. Finally we applied multiple constraints on every two candidate pairs to further remove incorrectly matched pairs. We were able to significantly reduce the number of iterations needed in the RANSAC algorithm, resulting in a much more efficient panorama stitching process. Experimental results demonstrated that (i) our corner matching is almost four times faster than the traditional RANSAC matching; (ii) panoramas generated from our algorithm feature a smooth transition in overlapping image areas and satisfy human visual requirements; and (iii) the preview speed of the generated panorama satisfies the real-time requirement commonly found in video panorama stitching.
