# Correction: Efficient Video Panoramic Image Stitching Based on an Improved Selection of Harris Corners and a Multiple-Constraint Corner Matching

**DOI:** 10.1371/annotation/eada5385-44a7-4a4f-aa1b-2194affdf310

**Published:** 2014-01-14

**Authors:** Minchen Zhu, Weizhi Wang, Binghan Liu, Jingshan Huang

Multiple funding organizations and grants were incorrectly omitted from the Funding statement. The Funding statement should read: 

“This research was supported by Natural Science Foundation of the Fujian Province, China (under Grant Numbers 2012J01263 and 2013J01186); Science and Technology Key Program of the Fujian Province, China (under Grant Number 2011Y0040); Science and Technology Innovation Platform Program of the Fujian Province, China (under Grant Number 2009J1007)."

